# Tracking the cell cycle origins for escape from topotecan action by breast cancer cells

**DOI:** 10.1038/sj.bjc.6600889

**Published:** 2003-04-15

**Authors:** G P Feeney, R J Errington, M Wiltshire, N Marquez, S C Chappell, P J Smith

**Affiliations:** 1 Department of Pathology, University of Wales College of Medicine, Heath Park, Cardiff CF14 4XN, UK; 2 Department of Medical Biochemistry, University of Wales College of Medicine, Heath Park, Cardiff CF14 4XN, UK

**Keywords:** topotecan, breast cancer, topoisomerase I, MCF-7, time-lapse microscopy, p53

## Abstract

The anticancer agent topotecan is considered to be S-phase specific. This implies that cancer cells that are not actively replicating DNA could resist the effects of the drug. The cycle specificity of topotecan action was investigated in MCF-7 cells, using time-lapse microscopy to link the initial cell cycle position during acute exposures to topotecan with the antiproliferative consequences for individual cells. The bioactive dose range (0.5–10 *μ*M) for 1-h topotecan exposures was defined by rapid drug delivery and topoisomerase I trapping. Topotecan caused pan-cycle induction and activation of p53. Lineage analysis of the time-lapse sequences identified cells initially in S-phase and G2, and defined the time to mitosis for cells originating from G2, S-phase and G1. Topotecan prevented all mitoses from S-phase cells and G1 cells (half-maximal effects at 0.14 *μ*M and 0.96 *μ*M, respectively). No dose of topotecan completely prevented mitosis among G2 cells, and at saturating doses of topotecan about half the cells of G2 origin continued dividing (the half-maximal effects was at 0.31 *μ*M). Overall, topotecan differentially targeted G1-, S- and G2-phase cells, but many G2 cells were resistant to topotecan, presenting a clear route for cell cycle-mediated drug resistance.

DNA topoisomerase I is a key target for anticancer therapy because of its crucial role in DNA metabolism and because it is often upregulated in cancer cells (O'Leary and Muggia, 1998; Bailly, 2000; Garcia-Carbonero and Supko, 2002). Topoisomerase I acts to relax the supercoils that accumulate in DNA during DNA replication (Wang, 1985) and transcription and does not show any strict cell cycle regulation (O'Leary and Muggia, 1998; Bailly, 2000; Garcia-Carbonero and Supko, 2002). During the relaxation reaction, a covalent intermediate between topoisomerase I and a 3′ single-stranded DNA (ssDNA) break is formed and this complex can become stabilized in the presence of the anticancer camptothecins (Hsiang and Lui, 1988; Shah and Schartz, 2001) to form cleavable complexes. Topotecan, a camptothecin derivative, is regarded as being S-phase selective (Bailly, 2000) because it is thought that cleavable complexes lead to dsDNA (double-stranded DNA) breaks during DNA replication (Hsiang and Lui, 1988; Kohn *et al*, 2000). Therefore, it is possible that many cells resist the effects of topotecan by failing to enter S-phase or replicate DNA during exposure to the drug (Shah and Schartz, 2001), although the true extent of this escape remains unclear. Understanding which cells are affected by camptothecin derivatives, and to what extent, is critical for the identification of potential mechanisms of cell cycle-mediated chemotherapy resistance (Shah and Schartz, 2001).

The S-phase selectivity of camptothecins (Gong *et al*, 1993) is consistent with the substantial reduction in their cytotoxic actions when cells are pretreated with the DNA polymerase inhibitor aphidicolin (Tsao *et al*, 1992; Wu *et al*, 2002). However, the use of replication inhibitors can also inhibit DNA polymerase-dependent DNA repair processes and can disrupt cycle traverse (Moore and Randall, 1987). In contrast, experiments involving short-term treatments with camptothecin derivatives where more than 80% of cells were killed (O'Connor *et al*, 1990,^1991^) suggest that the effects of camptothecins are not specific to S-phase cells. Indeed, camptothecin-induced DNA damage may be produced during transcription outside of S-phase as well as by DNA replication during S-phase (Mosesso *et al*, 2000), in keeping with a wider pan-cycle activity of topoisomerase I. Furthermore, reports of immediate G1 checkpoint activation following camptothecin exposure (Houser *et al*, 2001; Bozko *et al*, 2002) indicate the targeting of G1 cells. However, the eventual cell cycle consequences of topoisomerase I inhibition are difficult to link with the initial cell cycle position during the drug exposure period.

In this study, we used a high temporal resolution cell-tracking approach to measure the immediate pharmacodynamic effects of 1 h exposures to topotecan upon MCF-7 cells in each specific cell cycle phase in order to explore the origins of cell cycle-mediated resistance to the actions of topotecan. The phase-dependent consequences of topotecan treatment upon mitosis and apoptosis were measured without the confounding effects of cell synchronization or metabolic inhibition. Short-term exposures to topotecan were used in order to limit the cohort of cells undergoing DNA replication during drug exposure and to limit the confounding effects of pH-dependent topotecan inactivation (Chourpa *et al*, 1998). We present new evidence that topotecan directly targets cells in each cell cycle phase to prevent proliferation and rapidly induces a p53 response. In addition, we show that some G2 cells resist all acute doses of topotecan and continue to divide, providing a persistent route for cell cycle-dependent escape from topotecan. These pharmacodynamic responses to topotecan are correlated with topoisomerase I targeting by topotecan.

## MATERIALS AND METHODS

### Cell culture

Asynchronous MCF-7 cells, obtained from ECCC (Porton Down, UK), were cultured exclusively in Dulbecco's modified Eagles medium with 10% FCS at 37°C.

### Topotecan

Topotecan (Hycamtin®, SmithKline Beecham/Merck Pharmaceuticals, UK) was prepared in sterile water and the stock was stored at −80°C until required. MCF-7 cells were seeded at 3 × 10^4^ per well in standard plastic six-well tissue culture dishes for 24 h prior to treatment with topotecan for 1 h at 37°C, 5% CO_2_, followed by two washes with medium. Controls for drug treatment were handled in an identical manner, but treated with only sterile water.

### Cell cycle analysis

Cell cycle distributions were determined by analysis of ethidium bromide labelled cells as described (Smith *et al*, 1999) using a FacScan flow cytometer (Beckton-Dickinson, Cowley, UK). Cell cycle distributions were deduced as described by Watson *et al* (1987).

### DNA content *vs* p53 levels

Flow cytometry semiquantification of intracellular p53 levels *vs* DNA content (propidium iodide staining) was performed similarly to the method described previously (Gong *et al*, 1993). The expression of p53 was detected by labelling the cells using the DO-1 antibody (a pan-tropic anti-p53 antibody specific for residues 21–25).

### Time-lapse microscopy

MCF-7 populations that had been exposed to topotecan and washed, as described above, were incubated on the stage of the microscope in a standard plastic six-well culture dish. The instrument comprised a Zeiss Axiovert 100 microscope (Zeiss, Welwyn Garden City, UK) fitted with a temperature regulating incubator system and CO_2_ supply (Solent Scientific, Portsmouth, UK). The motorised xy microscope stage was from Prior Scientific and the phase transmission images (× 10 objective lens) were captured every 20 min over 48 h (145 frames per field) using an Orca I ER charge-coupled device camera (Hammamatsu, Welwyn Garden City, UK). The camera, stage (xy) and focus (z) were PC computer controlled via AQM 2000 software (Kinetic Imaging, Wirral, UK). Tiff-format Images (512 × 512 pixels) were played back for analysis as movies using the AQM 2000 software.

Initially, the time-lapse image sequences were manually analysed by counting the start and end numbers of cells, and by manually recording the time and nature of proliferative events during the observation period. Mitoses were deemed to have added one cell to the total number, ploidy events to have not changed the cell number and all cell deaths (whether the cells detached or not) to have decreased the cell number by one. The data was converted so that cell number changes were expressed as a percentage change from the starting number of cells (which ranged, for the proliferation curves from 288 to 349 cells per drug dose). The percentage changes in cell number were plotted as a function of time following the end of the topotecan treatment. Multiple fields ensured that sufficient numbers of cells were monitored to achieve the required time resolution. The cell cycle was mapped onto the proliferation curves using the data from bromodeoxyuridine (BrdU) time-lapse analysis of control cell populations. As detailed in the Results section, the expected time window for first-cycle G2 divisions was 0–8 h after topotecan exposure, while that of G1 cells was 16–20 h. S-phase was expected at 8–16 h. The proliferation rates for different cell cycle phases after exposure to topotecan (0.5–10 *μ*M) were measured from the slopes of the proliferation curves for control cells, and dose–response curves for each cell cycle phase were plotted and curves fitted using the nonlinear difference of squares approach. The graph software, GraphPad Prism (GraphPad.com), generated *R*^2^ values for goodness of fit.

In order to identify the cells in S-phase during a topotecan exposure, the cells were pulse-labelled for 15 min with 40 *μ*M bromodeoxyuridine (BrdU) at 37°C for 15 min and washed twice in medium immediately prior to the 1 h drug treatment. The time-lapse observations were performed and then the cells fixed and immunocytochemically stained to detect the BrdU label as described by Bond *et al* (1996). Brightfield images of the BrdU-labelled cells (147–197 total cells, about 30% labelled) within the time-lapse recorded fields were collected. The brightfield images were used to locate the BrdU-labelled cells in the time-lapse image sequences. The labelled cells were backward tracked from 48 h back to 0 h to identify the behaviour of cells that had been in S-phase during the topotecan treatment. Lineage analysis of the BrdU-labelled S-phase cells mapped the timings of first and subsequent divisions arising from these cells. Subsequently, G1 and G2 cells were identified by the time of their mitoses, respectively, after or before S-phase mitoses and their lineages analysed.

### Immunoblotting

Whole-cell lysates were prepared and immunoblotted as described by Webley *et al* (2000). p53 was detected using the monoclonal DO-1 clone (Oncogene Research Products, Nottingham, UK). Serine-15 phospho-p53 was detected using the rabbit polyclonal 9284 (Cell Signaling Technology Inc., Hertfordshire, UK). Topoisomerase I extracts for band depletion assays were prepared differently (Mo *et al*, 2000). Topoisomerase I was detected using clone C-21.2 monoclonal antibody (Beckton-Dickinson Pharmingen, Oxford, UK).

### Topotecan uptake assays

Topotecan has a UV excitable chromophore, therefore topotecan uptake into MCF-7 cells was assayed by flow cytometry (MW and PJS, manuscript in preparation). The assays were performed by incubating pretrypsinised MCF-7 cells with 10 *μ*M topotecan in fresh medium with 20 mM HEPES. At each time point, signals derived from excitation at multiline UV (351–355 nm) were collected at FL-4 via a 530/30 filter. The UV fluorescence signals were acquired and analysed using CELLQuest software (Beckton-Dickinson Immunocytometry Systems, Cowley, UK). Topotecan uptake was corrected for control sample autofluorescence.

## RESULTS

### Topoisomerase I trapping by topotecan in MCF-7 cells is rapid, dose-dependent and readily reversible

**Figure 1:**
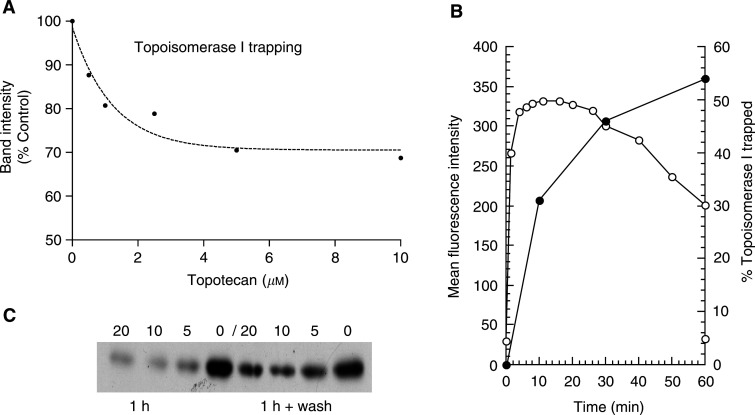
Topotecan rapidly enters MCF-7 cells to trap topoisomerase I. (**A**) Topotecan had trapped topoisomerase I into cleavage complexes, within 10 min, depleting the topoisomerase I bands, as detected by immunoblot. Topoisomerase I band depletion was dose dependent as demonstrated in a plot of topoisomerase I band intensity *vs* topotecan dose after a 10 min incubation period. (**B**) The intracellular topotecan concentration (○) and topoisomerase I trapping (•) during a 1 h incubation of MCF-7 cells with 10 *μ*M topotecan are compared. The maximal intracellular drug concentration was achieved within 10 min as measured by flow cytometry (mean fluorescence intensity, channel number). Intracellular topotecan concentrations declined over the following 50 min. In contrast, topoisomerase I trapping increased over a 1 h incubation with 10 *μ*M topotecan. (**C**) Immunblot topoisomerase I band depletions following 1 h incubations at 0–20 *μ*M are shown before (lanes 1–4) and immediately after washing twice (lanes 5–8) with medium to remove the topotecan. The dose of topotecan to which each of the samples had been exposed is indicated in *μ*M.

In order to measure the pharmacokinetics of the actions of short exposures to topotecan in MCF-7 cells, the dose relation for the trapping of topoisomerase I, the intracellular drug concentration and the reversibility of topoisomerase I trapping were examined. The pulse exposure regimen was chosen so that the perturbing effects of the relatively unstable drug and the discreet responses of the cells could be resolved with the minimum of cycle traverse during the treatment period. These data defined the bioactive dose range for topotecan in MCF-7 cells for the time-lapse experiments. Topoisomerase band depletion Western blots were performed to measure the topotecan dose relationship with topoisomerase I trapping. MCF-7 nuclei were extracted to yield only the topoisomerase I that had not been trapped into cleavable complexes by topotecan, such that trapping depleted the immunoblot topoisomerase I band. A plot of the ‘band depletion’ of topoisomerase I, following exposure to topotecan for 10 min is shown in Figure 1A

. Topoisomerase I was trapped into cleavable complexes rapidly and in a dose-dependent manner. Topoisomerase I trapping approached saturation at 5–10 *μ*M.

Topoisomerase I trapping increased with time such that 10 *μ*M topotecan had led to the trapping of 54% of the intracellular topoisomerase I after 1 h (Figure 1B). In contrast to the progressive trapping of topoisomerase I with topotecan exposure, detection of the intracellular topotecan levels (both active lactone and inactive hydroxy acid forms) by flow cytometry revealed that the topotecan (10 *μ*M) rapidly entered cells, and the maximal topotecan concentration was achieved within 10 min (Figure 1B). The intracellular topotecan concentration then diminished to approximately 65% of the maximum level by the end of the 1 h exposure. This fall was probably a consequence of lactone-ring hydrolysis of topotecan (Chourpa *et al*, 1998) leading to a net decline in the concentration of lactone–topotecan available for cell entry. Importantly, cells that had been washed twice following the 1 h exposure rapidly decreased their topotecan content and had returned to untreated control levels by 30–40 min, confirming that, within the limits of detection, no drug persisted following the treatments (data not shown). When MCF-7 cells were exposed to topotecan for 1 h, the topotecan washed away and the cells immediately extracted for topoisomerase I, there was a clear reduction in enzyme trapping following washing, indicating that cleavable complexes are rapidly removed when the topotecan is removed from the external medium (Figure 1C). These data are consistent with the rapid pharmacodynamic action of topotecan in MCF-7 cells and the rapid reversibility of cleavable complexes when topotecan is removed (Kohn *et al*, 2000).

### Time-lapse analysis of the effects of topotecan on cell proliferation

**Figure 2:**
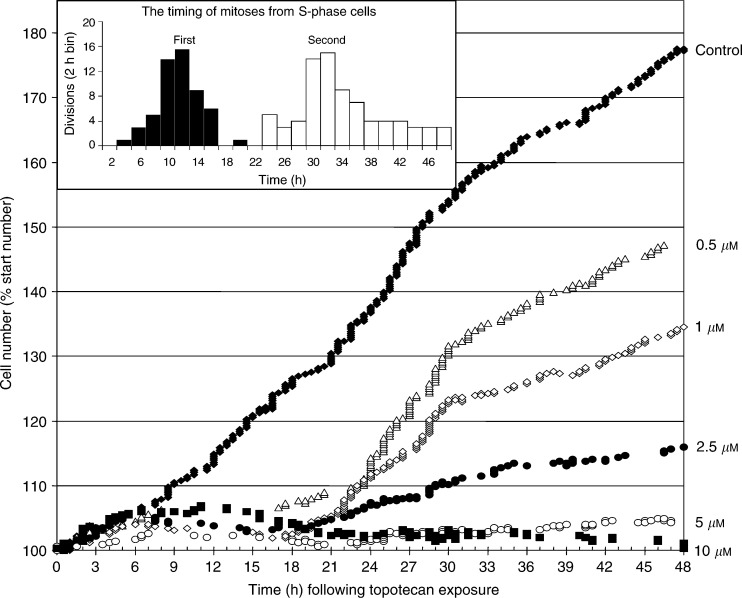
The dose dependency of the effects of 1 h topotecan exposures upon MCF-7 cell population expansion curves. The typical effects of 1 h exposures to topotecan upon the numbers of MCF-7 cells in cohorts of 288–349 cells are shown. The cell population sizes are expressed as the percentage of the size of each culture at time=0 h, the time immediately following the end of the topotecan exposure period. Each proliferative event is plotted as a single symbol, such that if few events occurred during a time period, gaps appear in the plot. The time-lapse observations were made at 20 min intervals. Control=(⧫); 0.5 *μ*M=(△); 1 *μ*M=(◊); 2.5 *μ*M=(•); 5 *μ*M=(○); 10 *μ*M=(▪). Inset: the timing of mitoses for untreated BrdU-labelled cells. In the histogram, the timings of first divisions (black filled bars) directly from BrdU-labelled S-phase cells and the second divisions (white bars) from the daughters of those BrdU-labelled S-phase cells are depicted.

Time-lapse proliferation analyses were used to investigate the effects of topoisomerase I inhibition by a discreet 1 h pulse exposure to topotecan. Cell population expansion curves consisted of plots of the number of cells expressed as a percentage of the number of cells immediately following the topotecan exposure (Figure 2

). The slopes of these graphs indicate the rates of change in cell numbers over the 48 h period of observation. Overall, MCF-7 cells exposed to topotecan (0.25–2.5 *μ*M) experienced an immediate slowing of their mitotic rates with cessation of proliferation at about 8 h, followed by dose-dependent proliferative recoveries beginning around 16 h after the topotecan exposure ended. There was little residual proliferation among cells exposed to 5 or 10 *μ*M topotecan. Flow analysis confirmed that proliferative recoveries occurred, with resumption of ‘normal’ cell cycle distributions at 24–48 h for cells exposed to 1 *μ*M topotecan, but not 10 *μ*M topotecan. Each individual event was plotted separately, such that the spacing of the points also indicated the rate of event occurrence, with larger spaces (gaps) in the plots at times where very few proliferative events occurred. In particular, the gaps between 8 and 16 h following topotecan treatment indicated that cells expected to enter mitosis during this period did not, while the downward trend in cell numbers over this period indicated that some cell loss (deaths) occurred.

### BrdU labelling of S-phase cells allows the initial cell cycle position of each cell to be deduced

To identify the cells that were in S-phase during the topotecan exposure period, MCF-7 cells were pulse labelled with BrdU for 15 min immediately before the topotecan exposure and time-lapse observation. BrdU prelabelling did not alter MCF-7 growth (data not shown). Backward tracking identified the cells were in S-phase during the topotecan exposure. There were 58 control cells (out of 197 total) that were in S-phase during the drug treatment period and 55 of these cells underwent mitosis. The timing of mitoses for the cohort of untreated MCF-7 cells that were in S-phase (first and second divisions) during the topotecan exposure period are plotted as a frequency histogram (inset Figure 2). The S-phase cell mitosis period was defined as the time-window during which the S-phase mitosis rate exceeded 50% of its maximum. Therefore, the first divisions of S-phase cells were defined as occurring 8–16 h after the treatment period ended and the divisions of their daughters (second divisions) at 28–36 h. The expected time window for first-cycle G2 divisions was 0–8 h after topotecan exposure, while that of G1 cells was after the first-cycle S-phase divisions, but >8 h before the second cycle S-phase divisions, that is, 16–20 h. Confirmation of this timing came from analysis of the distribution of second divisions from the G2 cells at 22–30 h after the drug treatment period. The duration of S-phase was 8 h, a complete cell cycle was approximately 20–22 h, G2≈8 h and G1≈6 h.

### Single-cell analysis allows the quantification of the differential effects of topotecan on G1, S and G2 phases

**Figure 3:**
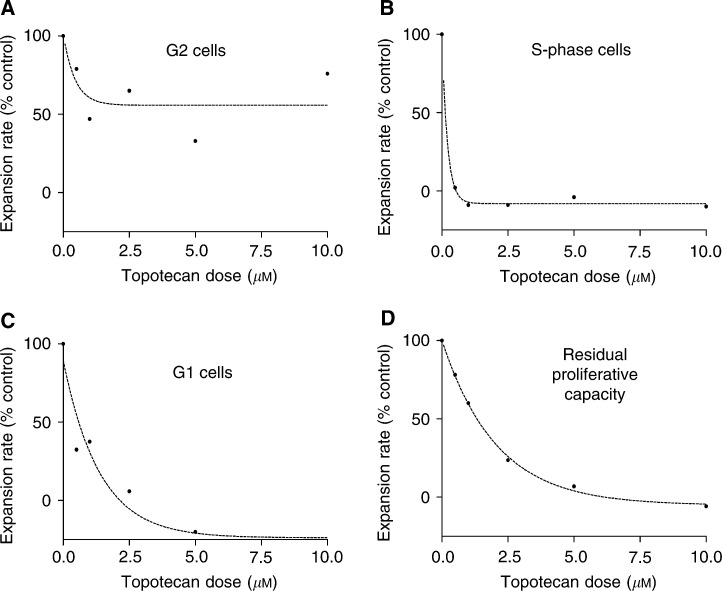
Dose–response curves for the effects of topotecan upon G2, S-phase and G1 cells, respectively, are shown. Based upon the expected mitosis rate, the effects of 1-h incubations with topotecan (0–10 *μ*M) upon mitosis rates for (**A**) G2 cells (*R*^2^=0.56) (**B**) S-phase cells (*R*^2^=0.99) and (**C**) G1 cells (*R*^2^=0.93) are shown. The subsequent residual proliferative capacity of the whole-cell populations after all first cycle divisions is shown in (**D**). Estimates of maximal decrease in mitosis rate (response, *M*_max_) and the topotecan dose required to achieve half-maximal response *D*_1/2_ were made from curves fitted to the data points: G2 *M*_max_=55.76%, *D*_1/2_=0.31 *μ*M; S-phase *M*_max_=−8.1%, *D*_1/2_=0.14 *μ*M; G1 *M*_max_=−23.9%, *D*_1/2_=0.96 *μ*M.

Identification of the time periods when G2, S-phase then G1 cells would be expected to deliver to mitosis, derived from unperturbed untreated control cell data meant that the mitosis rates for cells exposed to topotecan (0.5–10 *μ*M) could be measured separately for G2, S-phase and G1 originating cells (Figure 3

). The cells that were in G2 during the drug treatment period were prevented from reaching mitosis by topotecan in a dose-dependent manner (Figure 3A), with the expansion rate following higher doses of topotecan being reduced to approximately half that of untreated controls. Following exposure to any topotecan concentration (0.5–10 *μ*M), the G2 cells were not completely prevented from entering mitosis. The relatively noisy dose-response curve (goodness of fit *R*^2^=0.55) during this period reflects the relative paucity of mitoses. Lineage analysis of G2 cells (from a BrdU-labelled cohort) that had been exposed to topotecan was limited because the topotecan-treated G2 cells that failed to divide during 0–8 h after the drug treatment period could not be identified. However, lineage analysis of the 22 escaping G2 cells revealed that following 1 *μ*M topotecan exposure the number of daughter cells undergoing a second-round mitosis (11) was closely matched by the number dying (10), so that second-generation events arising from G2 cells caused no net change in cell number (Figure 2). This contrasts with the MCF-7 control populations where only two or three deaths would be expected among cells from all observed cells. Importantly, following exposure to topotecan (0.5–10 *μ*M), the surviving second-generation cells continued to divide providing a route by which some G2 cell lineages might survive the cytotoxic actions of topotecan.

When the S-phase cells would be expected to divide, 8–16 h after the drug treatment period, topotecan (0.5–10 *μ*M) resulted in a net loss in cell number (Figure 3B). Interestingly, this S-phase targeting remained saturated even after exposure to low doses of topotecan (100 nM for 1 h, data not shown). BrdU lineage analysis of the time-lapse data allowed the direct identification of S-phase cells, such that the complete cessation of mitosis from S-phase cells following exposure to topotecan (0.5–10 *μ*M) could be directly observed. For example, following exposure to 1 *μ*M topotecan all 68 S-phase cells (out of a total of 199 cells) failed to enter mitosis.

Topotecan reduced the rate of mitosis among G1 cells in a dose-dependent manner. As with S-phase cells, the maximal effect was that topotecan prevented all divisions from G1 cells (Figure 3C), but half-maximal responses required a higher concentration of topotecan (0.96 *μ*M) than S-phase cells (0.14 *μ*M). Therefore, G1 cells were approximately seven-fold less sensitive to topotecan than S-phase cells. The targeting of G1 cells by topotecan was demonstrated by their failure to enter mitosis at 16–20 h. It remains uncertain from the time-lapse data alone whether G1 cells exposed to 0.5–2.5 *μ*M topotecan were delayed or completely prevented from entering mitosis. However, following exposure to 5–10 *μ*M topotecan, very few cells at all entered mitosis during the 48 h observation period. The mitosis rates 20–48 h after topotecan exposure (Figure 3D) were reduced in a dose-dependent manner, reflecting the net effects of the differential cell cycle targeting by topotecan upon the overall residual proliferative capacity of the MCF-7 cells.

### Topotecan causes the multipoint slowing of cell cycle traverse, with G2 arrest

Although the single-cell analyses have shown that topotecan prevented G1- and S-phase cells from reaching mitosis, it remained unclear whether G1- and S-phase cells continued to traverse the cell cycle. Previously, cell cycle analysis of the effects of camptothecin derivatives has shown them to generate transient S-phase peaks followed by G2 arrest (Tsao *et al*, 1992). The effects of topotecan (1 and 10 *μ*M for 1 h) upon the cell cycle distribution 17 h later was measured. This time period was chosen such that cells in G1 during the drug treatment period should, if unperturbed, have traversed the cell cycle and reached mitosis. At 17 h, the cell cycle distributions were as follows: control G1 46.2%, S 41.3% and G2 12.5%; 1 *μ*M topotecan treated: G1 28.6%, S 32.5%, G2 38.8%; 10 *μ*M topotecan: G1 32.8%, S 32.3%, G2 34.8%. When MCF-7 cells were exposed to topotecan, there was a substantial accumulation of cells in G2, confirming that cells are prevented from entering mitosis by topotecan. It was also clear that there was significant emptying of the G1 fraction, indicating that G1 cells continue to traverse the cell cycle following exposure to topotecan. The traversing G1 cells would have passed into S-phase, so the reduced S-phase fraction 17 h after topotecan treatment demonstrated that S-phase cells, too, continued to traverse the cell cycle. In the time-lapse sequences, it was observed that most nondividing cells continued to increase in size, such that cells exposed to 10 *μ*M topotecan did not expand in numbers but did increase in surface area to cover the culture surface during the 48 h observation period.

### Topotecan exposure leads to rapid stress responses in all cell cycle phases

**Figure 4:**
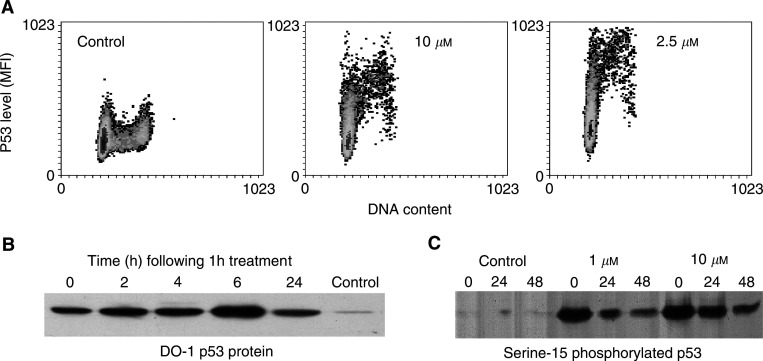
The levels of p53 across the cell cycle are increased rapidly by topotecan exposure. These changes are shown over time, following 1 h exposures to topotecan which ended at *t*=0 h. (**A**) Density plots of p53 *vs* cell cycle at the overall peak p53 levels (at 3 h) are shown, following exposure to 0, 2.5 and 10 *μ*M topotecan. (**B**) Western blot to show the levels of p53 protein in MCF-7 cells at 0–24 h following exposure to 10 *μ*M topotecan for 1 h. The increased p53 levels persisted at 48 h (not shown). (**C**) A Western blot showing that p53 was phosphorylated at serine-15 indicating activation of the protein following exposure to topotecan for 1 h. The time in hours following exposure to 0 *μ*M (control), 1 and 10 *μ*M topotecan is shown above each band.

The pan-cycle activation of p53 in response to 20 *μ*M camptothecin has been recently described (Houser *et al*, 2001), indicating that pan-cycle genotoxic stress is induced by short exposure to camptothecin, albeit against an apoptotic background. The activation of the tumor suppressor protein p53 plays an important, though not essential, role in the activation of the responses of the genotoxic stress pathways (Schartz and Rotter, 1998). The activation of p53 may even provide a means to distinguish the consequences of genotoxic dsDNA break induction from less-toxic ssDNA break formation (Nelson and Kastan, 1994). The elevation of p53 protein levels was detected, by flow cytometry, using the DO-1 antibody, immediately following a 1 h exposure to topotecan. The p53 levels peaked at 3 h post-treatment (Figure 4A

) and had diminished rapidly, back almost to control levels by 12 h (not shown). However, we have consistently observed that a 1 h exposure to 2.5 *μ*M topotecan caused a greater elevation of p53 levels than exposure to 10 *μ*M topotecan (Figure 4A). Cells in S-phase and G2/M had the highest p53 levels following topotecan exposure. Among the G0/G1 fraction, p53 was induced in only a subset of the cells (Figure 4A) giving an elevated mean fluorescence intensity.

In contrast to the results seen by flow cytometry, Western blotting of extracts of cells exposed to 10 *μ*M topotecan showed that the levels of p53 peaked at 6 h and remained high over the 48 h observation period (Figure 4B). This suggests epitope masking of the DO-1 antigen (for flow cytometry) following the induction of p53, indicating that the localization and functions of p53 may have been changed by exposure to topotecan. Further evidence of that topotecan caused a stress response was that p53 was phosphorylated at serine-15 following exposure to 1 and 10 *μ*M topotecan for 1 h and remained activated during the 48 h observation period (Figure 4C).

## DISCUSSION

In order to investigate cell cycle-mediated resistance to topotecan (Shah and Schartz, 2001), we have used a single-cell tracking approach to the analysis of high temporal resolution time-lapse sequences. For the first time, we have shown that the individual cell-cycle position of cell at a given period in time maybe linked with the proliferative outcome for that same cell without perturbation by metabolic inhibitors or synchronization. The study reveals the significant differential actions of topotecan across the cell cycle resulting in rapid induction of stress responses and failure to engage mitosis. The data presented shows that topotecan induced cellular stress is immediate in S-phase, G1 and G2 cells and that, therefore, replicon collision with cleavable complexes does not represent the sole mechanism of topotecan-induced stress. Here, we identify cells in G2 that are capable of escaping topoisomerase I inhibition, with major implications for the design rationale for the scheduling of topotecan anticancer therapy.

MCF-7 cells were chosen for this investigation given the widespread use of the cell line in breast cancer research and because they are relatively resistant to rapid DNA damage-induced apoptosis, in part because of deficiencies in caspase 3 (Janicke *et al*, 1998) and p73 (Zhu *et al*, 2001). Reduced early commitment to apoptosis eliminated the prejudicial effects of the loss of cells to a ‘dying fraction’. The pharmacokinetic profiles of topotecan with MCF-7 cells were investigated in order to determine the relation between topotecan uptake and cleavable complex formation and to define the bioactive dose range for MCF-7 cells. Topotecan rapidly entered MCF-7 cells to trap topoisomerase I into cleavable complexes. Interestingly, topoisomerase I trapping was both topotecan dose- and time-dependent, while the intracellular topotecan concentration peaked within 10–12 min, and then declined. Our experiences with other cell lines is that total topotecan never reaches a steady-state equilibrium (Smith *et al*, 2002 and manuscript submitted). Lactone–topotecan can cross cell membranes, while the net charge on hydroxy acid topotecan prevents cross-membrane diffusion (Gabr *et al*, 1997). Initially, the proportion of external lactone–topotecan is high and this drives the cellular accumulation of topotecan in combination with high-affinity intranuclear lactone–topotecan binding, resulting in the intracellular topotecan concentration being greater than in the extracellular medium, peaking after 10–12 min. Subsequently, the total cellular drug concentration falls as lactone–topotecan diffuses back out of the cell during a continual equilibration between the high-concentration intracellular lactone–topotecan pool and the diminishing extracellular topotecan pool subject to lactone ring hydrolysis (Smith *et al*, 2002 and manuscript submitted).

In this study, trapping of topoisomerase I was progressive with time during a 1 h incubation with 10 *μ*M topotecan, in the face of a declining intracellular topotecan concentration. Therefore, it is not the intracellular topotecan concentration (active plus inactive drug) *per se* that determines the targeting of the enzyme and the levels of cleavable complex formation. Indeed, it seems that the presence of topotecan in a cell leads to progressive topoisomerase I trapping that is irreversible until the drug is removed from the surrounding medium and it may be that there is some localization of topotecan at topoisomerase I sites that determines the levels of cleavable complex formation. The exact kinetics of cleavable complex formation and removal are complex because of the instability of active lactone topotecan and because cleavable complexes may not be directly reversed, but rather removed by enzyme action (Yang *et al*, 1996) and the DNA damage subsequently repaired.

It was found that topotecan (0.5–10 *μ*M) was active against all S-phase cells and acts in a dose-dependent manner against G1 and G2 cells. Crucially, it was found that position in the cell cycle during topotecan exposure alters the sensitivity to topotecan and the net proliferative outcome. These observations are in keeping with the previous reports that camptothecins may cause DNA damage outside of S-phase (O'Connor *et al*, 1990,^1991^; Mosesso *et al*, 1999,^2000^) and reveal the surprisingly large extent of non-S-phase targeting by these topoisomerase I inhibitors. Exposure of MCF-7 cells to topotecan resulted in the rapid elevation of p53 levels in all cell cycle phases. Not all of the G0/G1 (2*n*) cells showed p53 induction; however, time-lapse analysis of the control cell populations indicated that around 30% of an untreated MCF-7 cell population did not divide over a 48 h observation period, it is probable that many of the nonresponsive 2*n* cells were in G0. Overall, the p53 induction data provided an indication that topotecan caused rapid genotoxic stress to cells in G1-, S- and G2-phases and this showed that topotecan acted immediately in the non-S-phase cells confirming that DNA replication was not a prerequisite for topotecan-induced cellular stress in non-S-phase cells. Activation of p53 by phosphorylation at serine-15 was also rapid and persisted for 48 h after exposure to 1 or 10 *μ*M topotecan for 1 h.

An increasing proportion of G1 cells was arrested with increasing dose of topotecan, suggesting heterogeneity in the sensitivity of these cells to topotecan. Surprisingly, the G1 cells proved only seven-fold less sensitive to topotecan compared to S-phase cells. The fate of the G2 cells was more complex. Interestingly, whatever the dose of topotecan (1 h), not all of the G2 cells were prevented from undergoing mitosis. It is important to note that while many G2 cells were prevented from entry to their first mitosis by topotecan, others resisted its effects and divided. It was found that the daughter cells from G2 cells were subject to abnormally high levels of apoptosis, but importantly some G2 daughters were left to continue to subsequent divisions. Single-cell analysis therefore showed that G2 cell lineages could survive topotecan-mediated cytotoxicity because of their cell cycle position, indicating that, in response to topotecan, caspase 3-independent apoptosis may be triggered only in cells that have subsequently passed through mitosis. This is a strong evidence that cell cycle-mediated resistance to camptothecin derivatives and perhaps topoisomerase I inhibitors in general can arise from G2 cells.

Analysis of the cell cycle perturbations as a result of exposure to topotecan complemented the time-lapse data and showed that topotecan caused cellular stress, leading to G2 checkpoint activation. To study the cell cycle dependency of the effects of topotecan, single 1 h pulse exposures were used to minimise the extent of ongoing cycle traverse during the treatment period. It was found that topotecan slowed G1- and S-phase traverse, with a significant cohort of cells accumulating in S-phase before a later marked G2 arrest. Consistent with previous observations, we confirmed that continuous exposures to topotecan led to slowing of cell cycle traverse for G1- and S-phase cells, with accumulation of cells at G2.

These results have implications for the rational scheduling and combination of topotecan in anticancer therapy. Currently, the practice is to deliver longer-term lower-dose topotecan in order to target each tumour cell as it is recruited into S-phase (Herben *et al*, 1996; Garcia-Carbonero and Supko, 2002). However arrested and residual slow cycling cells would be expected to avoid such recruitment (Shah and Schartz, 2001). In this study, we have shown that non-S-phase cells can be targeted by topotecan, therefore it is possible that non-S-phase cells, when targeted suboptimally may be delayed from reaching their optimal window for exposure to topotecan in a subsequent S-phase. The targeting of such escaping cells, particularly those of G2 origin may be a useful concept in the rational development of topotecan schedules, particularly in combination with other therapies.
